# A comparison of clinical characteristics between adolescent males and females with eating disorders

**DOI:** 10.1186/s12888-015-0419-8

**Published:** 2015-03-11

**Authors:** Elisabeth Welch, Ata Ghaderi, Ingemar Swenne

**Affiliations:** Division of Psychology, Department of Clinical Neuroscience, Karolinska Institutet, Stockholm, Sweden; Department of Women’s and Children’s Health, Uppsala University, Uppsala, Sweden

**Keywords:** Clinical characteristics, Adolescent, Male, Anorexia nervosa, Eating disorders

## Abstract

**Background:**

Eating disorders (ED) are serious disorders that have a negative impact on both the psychological and the physiological well-being of the afflicted. Despite the fact that ED affect both genders, males are often underrepresented in research and when included the sample sizes are often too small for separate analyses. Consequently we have an unclear and sometimes contradictory picture of the clinical characteristics of males with ED. The aim of the present study was to improve our understanding of the clinical features of adolescent males with eating disorders.

**Methods:**

We compared age at presentation, weight at presentation, history of significantly different premorbid weight and psychiatric (Attention Deficit Hyperactivity Disorder (ADHD)) and somatic comorbidity (celiac disease and diabetes) of 58 males to 606 females seeking medical care for eating disorders at the Children’s Hospital in Uppsala, Sweden during the years 1999–2012. As all boys were diagnosed with either AN or Other Specified Feeding or Eating Disorder (OSFED) atypical AN, the age and weight comparisons were limited to those girls fulfilling the diagnostic criteria for AN or OSFED atypical AN.

**Results:**

There was no significant difference in age at presentation. Differences in weight at presentation and premorbid weight history were mixed. A significantly higher percentage of males had a history of a BMI greater than two standard deviations above the mean for their corresponding age group. As well, there was a higher prevalence of ADHD among the males whereas celiac disease and diabetes only was found among the females.

**Conclusions:**

A better understanding of the clinical characteristics of males with ED at presentation should improve our ability to identify males with ED and thereby aid in tailoring the best treatment alternatives.

## Background

Eating disorders (ED) are serious disorders that have a negative impact on both the psychological and the physiological well-being of the afflicted. The mortality rate associated with ED is high; for example, AN has the highest mortality rate of any psychiatric disorder [1], although recent studies indicate a trend towards a decrease in rate (e.g., [2]), likely mirroring earlier detection and improved care of these patients. Suicide attempts are also frequent; in fact, Damstedt, Petersen, Bilenberg and Hörder [3] found suicidal behavior in 40% of adolescents with ED. A large, nationally representative face-to-face survey in the United States (*n* = 9282) estimate the lifetime prevalence for anorexia nervosa, bulimia nervosa and binge eating disorders to be close to 6% [4]. When including sub-threshold binge eating disorder as well as any binge eating, the lifetime prevalence rises above 10%. A recent study of the rates of ED discovered that 16% of patients aged 14–20 years presenting at the emergency department screened positive for an eating disorder [5].

Furthermore, eating disorders have traditionally been thought to affect mainly females. As recently as four decades ago there was a debate as to whether or not males could have eating disorders at all, especially AN (e.g., [6]). Although there is a consensus today that ED do not discriminate between genders, there are different opinions as to how common ED are among males. The American Psychiatric Association [7] suggests that 10% of individuals that present with AN are males. However, other studies report different and sometimes significantly higher male/female ratios. For example, Woodside et al. [8] report a male/female ratio of 1:2 for full or partial syndrome AN and 1:2.9 for full or partial syndrome bulimia nervosa (BN) in a community sample using the World Health Organization’s Composite International Diagnostic interview. In line with these higher numbers, a recent clinical study from Australia found that among children aged 5–13 years, as many as one quarter of those diagnosed with an early onset eating disorder were boys [9]. Similarly, Dooley-Hash et al. [5] found that 26.6% of adolescents and young adults that screened positive for an ED upon presenting at the emergency department were male.

Despite the fact that eating disorders affect both genders, and perhaps substantially more males than previously thought, Galusca and colleagues report that only 1% of the research articles on AN (in a PubMed search) concern males [10]. Not only are males often underrepresented in the literature, when they are included the sample sizes are often too small to be analyzed on their own or in comparison to females [11]. Thus it is not surprising that today, over three centuries after the first descriptions of ED by Richard Morton (which did include both genders), we are still far away from a clear picture of the clinical characteristics of males with ED. This point has been highlighted by Darcy [12] who stated that many of the current assumptions regarding males with ED do not rest on a solid empirical foundation.

There is evidence of many similarities between ED in males and females (e.g., [13,14]). Vandereycken and Van den Brouck [15] go as far as to state in their review article of AN in males that the characteristics of males are nearly indistinguishable from those of females. However, when looking at specific characteristics such as age and weight at initial assessment, premorbid weight, somatic comorbidity and psychiatric comorbidity the picture becomes more complicated and sometimes contradictory, making interpretation of the data difficult. Upon inspection of a number of ED studies with a minimum of 20 males (thus excluding single case reports, observational studies, opinion articles, and studies of individuals with syndromes that result in disordered eating), the following picture emerges.

Several studies suggest that there are no gender differences in age at presentation [13,16-18], although other studies found that males are older [19] or younger at initial presentation [15]. Findings regarding gender differences in weight at presentation are divided, with some research indicating no gender differences [13,20], and other studies showing males being relatively heavier [18,19,21,22]. The difficulty in comparing studies reporting weights is compounded by the fact that different studies used different measures (i.e. BMI, % of ideal weight or matched population mean weight). The picture for premorbid weight is similar with some researchers finding no gender differences [13] and others finding that males are more likely to have been overweight [18,19,21]. A lack of consensus also appears when looking at comorbidity, which is an important variable associated with increased mortality, poorer treatment outcome and higher risk of relapse. While some studies have found similar rates of psychiatric comorbidity in males and females [8,9,20,21], others found higher rates in males compared to females (e.g., [13,18]) and yet others found lower rates of comorbidity among males [2,23]. Looking specifically at attention deficit hyperactivity disorder (ADHD), a review article by Curtin, Pagoto and Mick [24] showed that five out of eight studies found an association between ADHD and ED or eating pathology in adolescents. Although ADHD has a male preponderance in the general population [25], half of the studies included in the review were based solely on female samples and one of the studies failing to find an association was based predominantly on males. This pattern of mixed results calls for further studies elucidating the occurrence and nature of ADHD symptoms among males with ED.

Somatic comorbidity in males has not received much attention in research, but according to Erdur et al. [26], it plays an important role in the outcome of AN. In their 21-year follow up of female inpatients, 24.3% had a somatic comorbidity. Two specific somatic disorders that may be connected to ED are Type 1 diabetes and celiac disease. The connection of type 1 diabetes to ED has been explored in females (eg., [27]) whereas celiac disease has received little attention. Leffler, Dennis, Edwards George, & Kelly [28] described some complex ways in which celiac disease interacts with eating disorders, and discussed the clinical implications of these interactions for assessment and treatment of individuals with ED. Leffler et al. [28] argued that knowledge of both conditions is necessary for optimal care of patients with eating disorders or celiac disease. To the best of our knowledge no studies (with a minimum sample size of 20 males) have compared type 1 diabetes or celiac disease between genders in young individuals with ED.

Clearly, there are insufficient data to allow conclusions to be drawn regarding gender similarities or differences in ED. In order to accurately identify males with ED, clinicians need a clearer picture of the characteristics of ED in males. In addition, knowledge on gender dependent characteristics help identify potential differences in moderating variables affecting the outcome of the treatment for males versus females. Therefore there is a need to improve the understanding of the clinical manifestations of males with ED and to look at how males differ from females.

The aims of the present study are to 1) look at the age of presentation for males and compare to that of females; 2) look at the weight at presentation for males and to compare to that of females; 3) look at the premorbid weight history to see if males have a history of a significantly different premorbid weight compared to females, and; 4) look at both psychiatric (specifically ADHD) and somatic comorbidity, in the form of diabetes and celiac disease, and their possible gender differences. To this end, data analysis was performed on all patients seeking medical care for ED at the Children’s Hospital/Department of Child and Adolescent Psychiatry in Uppsala, Sweden, during the years 1999–2012.

## Methods

### Study group and procedure

The male study group consisted of 58 male patients that were referred to the Uppsala University Children’s Hospital/Department of Child and Adolescent Psychiatry for medical assessment of a diagnosed or suspected eating disorder in the time period from 1999–2012. They were compared with 606 female patients from the period 2004–2012. All participants and their respective guardians consented to participating in the study.

The protocol was approved by the ethics committee of the Faculty of Medicine of Uppsala University.

At initial contact with the clinic all patients were carefully assessed by a medical doctor specialized in pediatrics. A preliminary ED diagnosis, based on the Diagnostic and Statistical Manual of Mental Disorders 4th Edition (DSM IV) criteria was established and later confirmed following psychiatric assessment at the Eating Disorders Unit at the Department of Child and Adolescent Psychiatry. According to the DSM IV, the suggested weight criterion for AN in adults is a body weight less than 85% of that expected. However, a recent meta-analysis highlighted that this weight criterion is interpreted in a myriad of ways leading to quite different weight cut-offs for AN [29]. Thus, in accordance with the ICD-10 the criterion of a body mass index (BMI) below 17.5 was used in the current study (WHO, International Statistical Classification of Diseases and Related Health problems, 10th revision). In children and adolescents this corresponds to a BMI standard deviation score (*SDS*) below −2.00 [30]. Compared to BMI, BMI *SDS* offers the advantage of correcting for age. Furthermore, since eating disorder symptoms, as defined by the DSM IV, may not be verbally endorsed in young individuals but instead manifested in meal-related and other behaviors, care was taken to assess both symptoms verbalized by the patients and behaviors observed by parents [31,32]. All DSM IV eating disorder diagnoses were retrospectively re-categorized according to the DSM-5 criteria [33].

The clinic is the only specialized eating disorder service in the county and provides treatment for all adolescents <18 years with ED in the catchment area. At first assessment the patients were carefully screened for somatic disorders, and previously established psychiatric and somatic diagnoses were registered. Somatic assessment included a careful history of somatic symptoms, physical examination and blood sampling including serological markers for celiac disease.

Weight and height of all patients were collected at initial presentation. Premorbid weight and height were assessed by collecting growth charts from school health services. Only weights recorded by physicians or nurses were used in the study.

### Statistical analysis

Mean and standard deviations were calculated. In order to assess differences between the genders, independent samples *t*-test and Chi-square were conducted. When Levene’s test indicated unequality of variance, *t*-tests without assumed equal variances were performed. Effect sizes (Cohen’s *d*) were calculated when *p* < .05.

### Analysis of growth charts

From the growth charts, maximal recorded weight could be obtained. Weight loss was calculated as the difference between maximal recorded weight and weight at initial medical assessment. A pre-pubertal measurement of weight and height was obtained from the growth charts. This was usually measured around seven years age at a general health examination during the first year in school. Using growth charts, it was also possible to determine whether the pubertal growth spurt had started or whether the boys still followed the pre-pubertal trajectory. Body mass index (BMI) was calculated as weight/length^2^ (kg/m^2^). Measurements of weight, stature and BMI were also recalculated into standard deviation scores (*SDS*) [30]. As well, percent of weight for age was calculated.

## Results

Table 1 presents the clinically relevant characteristics of young male and female patients presenting with an ED at prepuberty, at top weight and at presentation.

**Table 1:** **Clinically relevant characteristics of young male and female patients presenting with AN or OSFED atypical AN at presentation, and corresponding characteristics at prepuberty, and at top weight**

	**Prepuberty**		**At top weight**		**At presentation**	
	**Boys**	**Girls**	**Boys**	**Girls**	**Boys**	**Girls**
	**n = 53**	**n = 491**	**n = 56**	**n = 562**	**n = 58**	**n = 541-572**
Age (years)	7.5 ± 0.99**^1^	7.24 ± 0.71	13.9 ± 1.7	14.2 ± 1.7	14.9 ± 1.6	15.2 ± 1.7
Weight (kg)	29.5 ± 7.9**^2^	26.6 ± 5.6	60.0 ± 16.8**^3^	55.0 ± 10.6	51.3 ± 9.6*^4^	48.8 ± 8.4
Height (cm)	128 ± 7	126 ± 9	167 ± 12	164 ± 3	170 ± 11***^5^	164 ± 8
BMI (kg/m^2^)	17.6 ± 3.2*^6^	16.7 ± 2.5	21.4 ± 5.1	20.7 ± 3.3	17.7 ± 2.1	18.1 ± 2.5
Weight SDS	0.86 ± 1.29*^7^	0.50 ± 1.11	0.78 ± 1.27*^8^	0.44 ± 1.11	−0.67 ± 1.06	−0.53 ± 1.12
Height SDS	0.27 ± 1.12	0.27 ± 1.07	0.39 ± 1.09	0.24 ± 1.11	0.18 ± 1.18	0.12 ± 1.15
BMI SDS	0.89 ± 1.38*^9^	0.51 ± 1.09	0.70 ± 1.47	0.39 ± 1.21	−0.99 ± 1.25	−1,00 ± 1.22
BMI SDS > 2.00	11 (20.1%)*	47 (9,6%)	11 (19,6%)*	44 (7,7%)	0	0
% weight for age	117 ± 25*^10^	111 ± 21	120 ± 31**^11^	110 ± 20	94 ± 16	93 ± 14
Weight loss (kg)	-	-	-	-	8,6 ± 7,3	6,9 ± 6.2
Duration (days)	-	-	-	-	347 ± 257	345 ± 279

SDS – standard deviation score.

BMI – body mass index.

Significance of difference between boys and girls by based on independent samples *t*-tests (for assumed equal and when applicable unequal variances) or Chi-square: *p < 0.05; **p < 0.01; ***p < 0.001.

Effect sizes: ^1^ *d* = 0.35, ^2^ *d* = 0.49, ^3^ *d* = 0.44, ^4^ *d* = 0.30, ^5^ *d* = 0.68, ^6^ *d* = 0.35, ^7^ *d* = 0.31, ^8^ *d* = 0.30, ^9^ *d* =0.34, ^10^d = 0,34, ^11^d = 0,49.

### Description of the samples and eating disorder categories

Ten (17%) of the males were classified as having Anorexia Nervosa (AN) and 48 males were classified as having an Other Specified Feeding or Eating Disorder (OSFED; atypical AN). No male patient received a diagnosis of BN or the bulimic subtype of OSFED. Among the females, 91 (15%) were classified as having AN, and 481 as having OSFED atypical AN. However, due to difficulties with the re-categorizing according to the DSM-5 criteria, it could not be determined if the remaining 34 of the girls should be classified as having BN, OSFED atypical BN or binge eating disorder (BED). According to the DSM-IV, eating disorder categories for the girls were as follows: 91 AN, 18 BN, 497 EDNOS (out of which 14 were classified as having bulimic subtype and two as having binge eating disorder). The mean age at presentation was 14.9 years (*SD* = 1.6) for males and 15.2 years (*SD* = 1.7) for females (*p* > 0.05), reflecting a small effect size (*d* = .18). No significant differences between the genders were found with respect to duration of illness, defined as time between the first symptom reported by either parent and/or patient and time of assessment.

### Age and weight

As all boys were diagnosed with either AN or OSFED atypical AN, the age and weight comparisons were limited to those girls fulfilling the diagnostic criteria for AN or OSFED atypical AN (see Table 1). At prepubertal measurement boys were significantly older, however only by an average of three months. The boys at prepuberty were also heavier and had higher BMI compared to girls. There were no differences in age at top weight, but boys were again heavier. A significantly higher percentage of males than females also had a BMI *SDS* over 2.00; 20.1% versus 9.6% (*χ*^2^(*df* = 1, *N* = 544) = 6.28, *p* ≤ .05) at prepuberty and 19.6% vs 7.7% (*χ*^2^(*df* = 1, *N* = 627) = 8.70, *p* ≤ .01) at top weight. At presentation the age was again similar between genders, and boys were heavier and taller than girls although there was no difference in BMI *SDS*.

### Comorbidities

Again only comparing the boys and girls with AN and OSFED atypical AN, psychiatric comorbidity in the form of ADHD was present in four boys (6.9%) and in six girls (1.0%). Somatic comorbidity in the form of type 1 diabetes or celiac disease was not found in any of the boys. Among the girls, 14 (2.4%) had biopsy confirmed celiac disease (of whom six were detected by laboratory screening at assessment), and six (1.0%) had type 1 diabetes mellitus.

Other psychiatric and somatic comorbidities identified at presentation, though not a specific aim of the present study, included among the boys six cases of other neuropsychiatric disorders (10.3%, two obsessive compulsive disorder, three autism spectrum disorder and one Tourette’s syndrome) nine cases of other somatic disorders (15.5%, one hypochondroplasia, two hypothyreosis, one ulcerative colitis, one pseudohypoparathyroidism, one Klinefelter’s syndrome, one Langerhans cell histiocytosis, one transplantation of teeth and one surgically corrected ventricular septal defect). The corresponding numbers among the girls were one case of other neuropsychiatric disorders in the form of obsessive-compulsive disorder (0.2%) and 12 cases of other somatic disorders (2.1%, four juvenile reumathoid arthritis, five hypothyreosis, one pubertas praecox, one cystic fibrosis and one partial deafness). Of note is the fact that among the girls with BN, OSFED atypical BN and BED there were no psychiatric or somatic comorbidity. Asthma and allergies were not uncommon but probably under-reported, especially if symptoms were temporary and not severe, which would preclude a correct estimate of prevalence.

## Discussion

The present study of 58 male adolescents with eating disorders is one of only about a dozen studies with adequate sample size and thus adequate statistical power to carry out meaningful statistical comparisons of clinical characteristics of eating disorders between genders. It is also one of the most recent studies, including data from 1999 to 2012 (data for females were collected from 2004 to 2012), which may help identify trends when compared with previous research. Below, we interpret our findings in light of the aims of the present study, which are to investigate gender differences in age and weight at presentation, premorbid weight, and both psychiatric and somatic comorbidity.

### Age at presentation

There was no difference in mean age at presentation. This finding is in agreement with some previous studies [13,16-18,34], but contrary to others [15] who found boys to be younger, and Sharp et al. [19] who found boys to be older at presentation. However, it should be noted that the actual mean ages at presentation for both males and females in the current study do differ from those reported in these other studies. For example, the mean ages of the samples in the study by Vandereycken and Brouke [15] were 17.2 for males and 20.1 for females, and the mean ages in the study by Crisp, Burns and Bhat [16] were 20.4 years for males and 20.8 years for females. Even higher mean ages were found by Guegen and colleagues [18], which were 26.6 years for males and 26.4 years for females, while Bramon-Bousch and colleagues [13] reported the highest mean age at presentation with 30.7 years for males and 29.3 years for females. Only Geist et al. [17] and Darcy et al. [34] found similar ages of presentation in their studies with 14.1 and 15.9 years, respectively, for males and 14.4 and 15.5 years, respectively, for females. The lower mean age of individuals in the current study is undoubtedly related to the fact that the study group consisted of patients referred to a children’s hospital (which do not accept patients above the age of 18 years), and thus one should be cautious when comparing the gender differences from this study to other studies that may include participants from a different age demographic.

### Weight at presentation and premorbid weight history

No significant gender differences with regard to weight in *SDS*, BMI or BMI *SDS* at presentation were found. These results are in line with some of the previous research [13,20]. With regards to premorbid weight history we found, in line with Sharp and colleagues [19], Fernández-Aranda and colleagues [21] and Gueguen et al. [18], that males had a higher prepuberty BMI, BMI *SDS* and weight measured in kilograms, as well as a higher top weight measured in kilograms and *SDS* but not as measured in BMI or BMI *SDS*. However, as many as one in every five males had a history of a premorbid BMI *SDS* greater than 2 both at prepuberty and at top weight, highlighting the need for clinicians to thoroughly investigate the previous weight history as our study indicates that males may present with a more drastic weight loss despite having a similar BMI *SDS* at presentation.

Again, it should be noted that it is difficult to compare differences in weight between studies since not all studies report the same measures (some report weight in kg other BMI and yet other percent of ideal weight). A universal reporting of weight would make comparison of results across studies simpler.

### Psychiatric comorbidity - ADHD

The present study found that a significantly larger proportion of males than females (6.9% versus 1.0%, respectively) at presentation had a previous diagnosis of ADHD. A recent systematic review and meta-regression analysis of the worldwide prevalence of ADHD [25] report an overall pooled prevalence of ADHD of 5.9% (95% *CI* = 5.01-5.56) with male gender being associated with significantly higher prevalence rates. Psychiatric comorbidity is associated with increased mortality, poorer treatment outcome and higher risk of relapse in ED [35] and therefore there is a pressing need for further studies in the area. ADHD is one of the important comorbid conditions, given the fact that it is a primary diagnosis among patients with ED when present, as opposed to depression, which although more prevalent is often secondary to ED. In addition, ADHD may explain some of the gender differences in ED since it is more prevalent in boys and it might affect the presentation of the ED symptoms or moderate the patients’ response to the treatment of ED.

### Somatic comorbidity – diabetes and celiac disease

None of the boys presented with, or were diagnosed with either type 1 diabetes or celiac disease whereas a small proportion of girls had either type 1 diabetes (1.0%) or celiac disease (2.4%). Data from the nationwide Swedish Childhood Diabetes Registry indicate that among children aged 0–14 years, the incidence among males is 46.7 per 100,000 and among females 42.1 per 100,000 [36].

The fact that half of the girls with celiac disease were diagnosed upon presentation is disconcerting; however, it is in line with research showing a significant number of undiagnosed cases of celiac in the population [37]. Interpretation of the absence of type 1 diabetes and celiac disease among the males in the present study cannot be made due to the small sample size. Still, the number of new identified cases among the females with celiac disease highlights the importance of screening for celiac disease during initial assessments for ED, especially since gastrointestinal symptoms may be absent and/or disguised by the ED.

There are two other interesting observations in the present study, although not part of the specific aims of the study. One is the high rate of other neuropsychiatric comorbidity (apart from ADHD) and somatic comorbidity (apart from diabetes and celiac) among the males, 10% and 15.5%, respectively, highlighting the need for further studies with more rigorous diagnostics. The second observation is that all of the males were classified with having either AN or OSFED atypical AN. No male patient and only 34 out of the 606 females received a diagnosis of BN, OSFED atypical BN or BED. This is in line with Nicholls, Lynn and Viner [38] who found in their study of the incidence of early-onset eating disorders that only 4% of the children (males and females) were identified with BN or other binge eating behavior. In a review article, Fairburn & Harrison [39] state that the proportion of males classified with having BN is uncertain, and therefore this issue requires further investigation. Additionally, BN usually has a later age of onset than AN and atypical AN (e.g., [39]) and given our recruitment base the low prevalence of BN diagnosis among the patients might be expected.

The findings from the present study should be evaluated within the context of several limitations and strengths. The study is based on data from a single, university hospital. On the other hand, the clinic is the only one in the region and thus all patients from Uppsala (the fourth largest city in Sweden) and the surrounding area are seen at the clinic adding to the representativeness of the sample. Apart from referrals from family physicians and school health services, parents can also contact the clinic directly. During the past 15 years, significant efforts have been made at the clinic to identify ED cases as soon as possible and provide structured treatment (behavioral family therapy) to avoid risk for a chronic path and entrenched cases. This may positively have affected the likelihood that youths suffering from eating disorders came into contact with the clinics, resulting in an enhanced generalizability of this study. One might argue that the difference in data collection periods for the males (1999 to 2012) versus females (2004 to 2012) poses a problem, however, no major changes in the healthcare system were undertaken during the time period and no changes were made in the referral routines. Another characteristic of male ED that has been increasingly documented among males is drive for muscularity. Thus, in future studies it would be of interest to look at drive for muscularity and compare gender differences among adolescents.

Although a relatively large number of male patients were included in the present study, replications with even larger sample sizes are needed to corroborate or falsify our findings. A longitudinal design with outcome data would be preferential. Through a better understanding of the clinical characteristics of males with ED at presentation we are better equipped to identify males with ED and thereby select the best treatment alternative. The present study can hopefully provide a piece in the complex puzzle surrounding this issue.

## Conclusions

The clinical characteristics of males with ED were not significantly different from females in several aspects such as age of onset and duration of illness. However, no male patient received a diagnosis of BN or the bulimic subtype of OSFED, or somatic comorbidity in the form of type 1 diabetes or celiac disease. A significantly higher percentage of males than females had a premorbid BMI *SDS* over 2.00, and a diagnosis of ADHD at presentation. Knowledge on the similarities and potential differences in clinical characteristics of males with ED at presentation might help professionals to pay attention to distinct features that improve identification, prevention and treatment of males with ED.

## References

[1] Sullivan PF (1995). Mortality in anorexia nervosa. A J Psychiatry.

[2] Lindblad F, Lindberg L, Hjern A (2006). Anorexia nervosa in young men: a cohort study. Int J Eat Disord.

[3] Damsted P, Petersen DJ, Bilenberg N, Horder K (2006). Suicidal behaviour in a clinical population of 12- to 17-year-old patients with eating disorders. Ugeskr Laeger.

[4] Hudson JI, Hiripi E, Pope HG, Kessler RC (2007). The prevalence and correlates of eating disorders in the National Comorbidity Survey Replication. Biol Psychiatry.

[5] Dooley-Hash S, Banker JD, Walton MA, Ginsburg Y, Cunningham RM (2012). The prevalence and correlates of eating disorders among emergency department patients aged 14–20 years. Int J Eat Disord.

[6] Kidd CB, Wood J (1966). Some observations on anorexia nervosa. Postgrad Med J.

[7] Diagnostic and Statistical Manual of Mental Disorders. 4th ed. Washington, DC: American Psychiatric Association; 1994.

[8] Woodside DB, Garfinkel PE, Lin E, Goering P, Kaplan AS, Goldbloom DS, Kennedy SH (2001). Comparisons of men with full or partial eating disorders, men without eating disorders, and women with eating disorders in the community. A J Psychiatry.

[9] Madden S, Morris A, Zurynski YA, Kohn M, Elliot EJ (2009). Burden of eating disorders in 5-13-year-old children in Australia. Med J Aust.

[10] Galusca B, Leca V, Germain N, Frere D, Khalfallah Y, Lang F, Estour B (2012). Normal inhibin B levels suggest partial preservation of gonadal function in adult male patients with anorexia nervosa. J Sex Med.

[11] Bulik CM, Berkman ND, Brownley KA, Sedway JA, Lohr KN (2007). Anorexia nervosa treatment: a systematic review of randomized controlled trials. Int J Eat Disord.

[12] Darcy AM (2011). Eating disorders in adolescent males: a critical examination of five common assumptions. Adolesc Psychiatry.

[13] Bramon-Bosch E, Troop NA, Treasure JL (2000). Eating disorders in males: a comparison with female patients. Eur Eat Disord Rev.

[14] Carlat DJ, Camargo CA, Herzog DB (1997). Eating disorders in males: a report on 135 patients. A J Psychiatry.

[15] Vandereycken W, Broucke S (1984). Anorexia nervosa in males. Acta Psychiatr Scand.

[16] Crisp AH, Burns T, Bhat AV (1986). Primary anorexia nervosa in the male and female: a comparison of clinical features and prognosis. Br J Med Psychol.

[17] Geist R, Heinmaa M, Katzman D, Stephens D (1999). A comparison of male and female adolescents referred to an eating disorder program. Can J Psychiatry.

[18] Gueguen J, Godart N, Chambry J, Brun-Eberentz A, Foulon C, Snezana M, Guelfi JD, Rouillon F, Falissard B, Huas C (2012). Severe anorexia nervosa in men: comparison with severe AN in women and analysis of mortality. Int J Eat Disord.

[19] Sharp CW, Clark SA, Dunan JR, Blackwood DHR, Shapiro CM (1994). Clinical presentation of anorexia nervosa in males: 24 new cases. Int J Eat Disord.

[20] Braun DL, Sunday SR, Huang A, Halmi KA (1999). More males seek treatment for eating disorders. Int J Eat Disord.

[21] Fernández-Aranda F, Aitken A, Badia A, Giménez L, Solano R, Collier D, Treasure J, Vallejo J (2004). Personality and psychopathological traits of males with an eating disorder. Eur Eat Disord Rev.

[22] Fichter MM, Daser C (1987). Symptomatology, psychosexual development and gender identity in 42 anorexic males. Psychol Med.

[23] Núnez-Navarro A, Agüera Z, Krug I, Jiménez-Murcia S, Sánchez I, Araguz N, Gorwood P, Granero R, Penelo E, Karwautz A (2011). Do men with eating disorders differ from women in clinics, psychopathology and personality?. Eur Eat Disord Rev.

[24] Curtin C, Pagoto SL, Mick E (2013). The association between ADHD and eating disorders/pathology in adolescents: a systematic review. Open J Epidemiol.

[25] Polanczyk G, de Lima M, Horta B, Biederman J, Rohde L (2007). The worldwide prevalence of ADHD: a systematic review and metaregression analysis. A J Psychiatry.

[26] Erdur L, Kallenbach-Dermutz B, Lehmann V, Zimmermann-Viehoff F, Köpp W, Weber C, Deter HC (2012). Somatic comorbidity in anorexia nervosa: first results of a 21-year follow-up study on female inpatients. Biopsychosoc Med.

[27] Nielsen S (2002). Eating disorders in females with type 1 diabetes: an update of a meta-analysis. Eur Eat Disord Rev.

[28] Leffler DA, Dennis M, George JBE, Kelly CP (2007). The interaction between eating disorders and celiac disease: an exploration of 10 cases. Eur J Gastroenterol Hepatol.

[29] Thomas JJ, Roberto CA, Brownell KD (2009). Eighty-five per cent of what? Discrepancies in the weight cut-off for anorexia nervosa substantially affect the prevalence of underweight. Psychol Med.

[30] Lindgren G, Strandell A, Cole T, Healy M, Tanner J (1995). Swedish population reference-standards for height, weight and body-mass index attained at 6 to 16 years (girls) or 19 years (boys). Acta Paediatr.

[31] Becker AE, Eddy KT, Perloe A (2009). Clarifying criteria for cognitive signs and symptoms for eating disorders in DSM-V. Int J Eat Disord.

[32] Bravender T, Bryant-Waugh R, Herzog D, Katzman D, Kriepe R, Lask B, Le Grange D, Lock J, Loeb K, Marcus M (2010). Classification of eating disturbance in children and adolescents: proposed changes for the DSM-V. Eur Eat Disord Rev.

[33] Diagnostic and Statistical Manual of Mental Disorders. 5th ed. Washington, DC: American Psychiatric Association; 2013.

[34] Darcy AM, Doyle AC, Lock J, Peebles R, Doyle P, Le Grange D (2012). The eating disorders examination in adolescent males with anorexia nervosa: how does it compare to adolescent females?. Int J Eating Disorders.

[35] Steinhausen H-C (2009). Outcome of eating disorders. Child Adolesc Psychiatr Clin N Am.

[36] Berhan Y, Waernbaum I, Lind TR, Möllsten A, Dahlquist G (2011). Thirty years of prospective nationwide incidence of childhood type 1 diabetes the accelerating increase by time tends to level off in Sweden. Diabetes.

[37] Myléus A, Ivarsson A, Webb C, Danielsson L, Hernell O, Högberg L, Karlsson E, Lagerqvist C, Norström F, Rosén A (2009). Celiac disease revealed in 3% of Swedish 12-year-olds born during an epidemic. J Pediatr Gastroenterol Nutr.

[38] Nicholls DE, Lynn R, Viner RM (2011). Childhood eating disorders: British national surveillance study. Br J Psychiatry.

[39] Fairburn C, Harrison P (2003). Eating disorders. Lancet.

